# Pressure injury risk assessment for palliative care patients in the acute hospital setting: a scoping review

**DOI:** 10.1186/s12904-025-01842-y

**Published:** 2025-07-25

**Authors:** Saroeun Ven, Paul Fulbrook, Adam Burston, Josephine Lovegrove, Sandra J Miles

**Affiliations:** 1 School of Nursing, Midwifery & Paramedicine, Faculty of Health Sciences, Australian Catholic University, 1100 Nudgee Road, Banyo, QLD 4014 Australia; 2 Nursing Research and Practice Development Centre, The Prince Charles Hospital, 627 Rode Rd, Chermside, QLD 4032 Australia; 3 National Health and Medical Research Council Centre of Research Excellence in Wiser Wound Care, Griffith University, Gold Coast, Chermside, QLD 4222 Australia; 4 School of Nursing, Midwifery and Social Work, The University of Queensland, UQ Centre for Clinical Research, Royal Brisbane & Women’s Hospital Campus, Building 71/918, Herston, QLD 4029 Australia; 5 Herston Infectious Diseases Institute, Metro North Hospital and Health Service, Herston, QLD 4006 Australia

**Keywords:** Acute care, Palliative care, Pressure injury, Risk assessment

## Abstract

**Background:**

Hospitalised palliative care patients are at risk of pressure injury. The development of pressure injuries causes physical and psychological distress for patients. Therefore, the prevention of hospital-acquired pressure injury is a nursing priority. The first step in prevention is conducting a risk assessment, which should be population-specific. In Australia, four palliative care phases guide care planning for appropriate clinical management of palliative care patients. In phases 1–3, the palliative patient cohort is based on acute care, where patients require medical treatment and/or symptom management, whereas Phase 4 refers to end-of-life care.

**Objectives:**

To review and analyse contemporary literature to determine what is known about pressure injury risk assessment for acute palliative care patients and identify which pressure injury risk assessment tools are most appropriate for this cohort.

**Methods:**

This scoping review was conducted according to Joanna Briggs Institute methodology. The search strategy was developed based on the Population-Concept-Context mnemonic. Studies of any design, articles and guidelines relating to pressure injury risk assessment in acute palliative care patients were included. Publications from 2002 to 2023 from Google Advanced Search, three grey literature and five nursing/health databases (Cumulative Index to Nursing and Allied Health Literature, MEDLINE, Scopus, Web of Science, EMBASE) were searched. The protocol was prospectively registered with Open Science Framework.

**Results:**

From 2,576 search results, 15 articles met the inclusion criteria. None reported the use of a pressure injury risk assessment tool designed specifically for acute palliative care patients. However, 20 pressure injury risk assessment tools/methods were identified. Furthermore, the definition of palliative care patients was inconsistent, and no articles clearly defined the differences between acute palliative care patients and those at end-of-life.

**Conclusions:**

The results of this review indicate a need to develop a new pressure injury risk assessment tool for acute palliative care patients that considers their specific risk factors. Further research is needed to address the knowledge gap relating to pressure injury risk assessment and prevention for hospitalised acute palliative care patients.

**Supplementary Information:**

The online version contains supplementary material available at 10.1186/s12904-025-01842-y.

## Introduction

The aim of palliative care is to improve the quality of life (QoL) of all individuals living with a life-limiting illness [1], not just those at end-of-life (EoL) [2]. The global demand for specialist palliative care is increasing steadily to meet the needs of an aging population and an increase in chronic degenerative and disabling health conditions (e.g., dementia, chronic obstructive pulmonary disease, osteoarthritis) [3, 4]. In Australia, palliative care phases (1: Stable, 2: Unstable, 3: Deteriorating, 4: Terminal), are used to describe the clinical status and care planning for palliative care patients [5–7]. Although, there is a fifth phase i.e., Bereavement, this relates to post-death support provided to family/carers [7]. In terms of patient care, phases 1 to 3 (Stable, Unstable, Deteriorating) describe acute medical care from a palliative care specialist, whereas phase 4 (Terminal) describes comfort care for individuals at EoL (death is likely within days [7]).

Palliative care patients admitted to an acute hospital setting require *acute* management and treatment with the goal of discharge back home or to residential settings [8]. Acute crisis in palliative care relates to clinical situations that arise due to underlying disease-specific and pathophysiological changes negatively impacting their QoL. To improve the QoL of acute palliative care patients, acute treatments are aimed at managing symptoms such as acute severe pain, nausea and vomiting, dyspnoea, airway obstruction, agitation and altered mental state, spinal cord compression, haemorrhage, and seizures [9–12]. However, during their admission, acute palliative care patients are at risk of developing hospital-acquired complications. In this context, hospital-acquired pressure injury (PI) is the most common type of ulcer experienced by palliative patients [13–15] due to their generally poorer clinical condition and decreased functional status [16, 17]. Subsequently, to optimise QoL, the prevention of PI is recognised as an essential element of holistic care [13, 14].

Each year, PI accounts for one of the highest reported hospital-acquired complications, resulting in a financial burden for hospitals (cost of treatment and excess length of stay) [16, 18, 19]. Its prevention is a clinical priority to prevent negative impacts on patients, including pain, psychological distress and reduced QoL [19–21]. The international PI guideline states that individuals receiving palliative care are a unique population with specific PI prevention and treatment needs [22]. Timely recognition of at-risk patients in this population may help nurses plan and deliver appropriate care to prevent adverse outcomes.

Pressure injuries are localised damage to the skin and/or underlying soft tissue, caused by or in combination with pressure, friction or shear [22]. In contrast, PIs associated with EoL (*terminal ulcers*) are described as being unavoidable [23], as they are related to failing organs [24–26]. Since the term Kennedy Terminal Ulcer (KTU) was introduced in 1989, the concepts of terminal ulcers, Skin Changes At Life’s End (SCALE), and skin failure have been established in the literature [23].

However, acute palliative care patients are not necessarily experiencing organ failure to the same degree as EoL patients and furthermore they have a distinctively different illness trajectory compared to other acute patients in general medical wards [27]. Therefore, the focus of this scoping review is on the prevention of PI that occurs during the acute palliative phases (Phases 1–3), as distinct from those occurring in patients at EoL.

To prevent PI, a key step is to assess the level of risk of individual patients based on their condition and treatment. While several PI risk assessment tools are available in various clinical settings, none have been designed specifically for acute palliative care patients, and minimal research is available to inform PI risk assessment decisions. A greater understanding of PI risk assessment is required to prevent hospital-acquired PI for patients in acute palliative care.

While acute palliative care is a term used clinically, it is not as evident in research literature, so there is a potential research gap relating to PI in acute palliative care patients. Scoping reviews map published literature to guide researchers and clinicians to identify and describe knowledge gaps, providing a valuable guide for future clinical practice and research [28]. To fully understand what is known about PI risk assessment for acute palliative care patients, the purpose of this scoping review was to map the existing research and explore the relevant literature pertaining to PI risk assessment tools within acute palliative care. The aim of this review was to identify and present the available evidence regarding PI risk assessment tools or scales used to assess the PI risk level of adult acute palliative care patients (Phases 1–3) in an acute hospital setting. The following questions are addressed:

1. What risk assessment tools or scales have been used to assess PI risk levels for adult palliative care patients, including acute palliative care patients but excluding those at EoL?
2. What are the characteristics and risk factors of adult palliative care patients, including acute palliative care patients, but excluding those at EoL, who develop PI?

## Methods

The Joanna Briggs Institute (JBI) methodology for scoping reviews [28] was used to guide the literature search, with the primary objective to map knowledge concerning PI risk assessment for acute palliative care patients. This methodology was derived from the frameworks of Arksey and O’Malley [29] and Levac et al. [30], refined to provide more clarity for each element required in a scoping review [28, 31]. Population, Concept, Context (PCC) was used to determine appropriate search terms, as it offered a broad yet structured review, avoiding a narrow focus and enabling a more comprehensive range of articles to be considered for inclusion [32].

A team approach was taken with co-authors established for the study selection and extraction process and a third person to act as an arbitrator [28, 32]. Health service librarians were consulted during the preliminary searches and assisted with developing and refining search terms. The reporting method for this scoping review was conducted according to the Preferred Reporting Items for Systematic Reviews and Meta-Analyses for Scoping Reviews (PRISMA-ScR) [33, 34].

### Protocol registration

An a priori protocol was developed for methodological rigour [35], outlining the objective, inclusion and exclusion criteria, and methods for conducting the scoping review [28], and was prospectively registered with the Open Science Framework (OSF) database [36].

### Eligibility criteria

The PCC mnemonic was used to guide the selection of eligibility criteria for this scoping review and was linked to the research aim and questions (see Table 1). To explore the breadth of the literature map and summarise the available evidence, the review included all types of primary and secondary research studies, discussion papers and expert opinions discussing palliative care patients (Phases 1–3). Parts of articles or research studies containing information or discussion of the topic of interest were included for example, studies reporting on palliative care generally, with subset data applicable to ‘acute palliative care’. In 2002, the World Health Organization broadened its definition of palliative care to include all individuals living with life-limiting illnesses [1]. Thus, year limiters from January 2002 to March 2023 were applied to the search strategy to represent contemporary palliative care settings and patient cohorts. Articles in languages other than English were excluded due to time constraints and resource limitations for translations. Where full text was unavailable, articles were excluded due to insufficient information for meaningful data extraction.

**Table 1:** Eligibility criteria

**Population**	Acute palliative care adults ≥ 18 years of age. Since the characteristics of palliative care patients who develop EoL-associated PI are arguably different to those in acute palliative care [57], articles focused solely on palliative care patients at EoL were excluded. Articles including acute and EoL patients were included with information on the inclusion criteria extracted for analysis and synthesis.
**Concept**	Any method of PI risk assessment conducted by nurses were included. The PI risk assessment could include, but not be limited to, a structured risk assessment tool or scale, clinical judgement alone, or a structured risk assessment in conjunction with clinical judgement.
**Context**	In the context of acute palliative care in an acute hospital setting, articles and studies discussing settings such as community, home, respite care, or settings that were not acute or tertiary settings were excluded.

EoL: end-of-life, PI: pressure injury

### Search strategy

The search strategy was developed based on the PCC and aimed to locate published studies, grey literature, clinical guidelines and reviews in five databases commonly used for nursing research: Cumulative Index to Nursing and Allied Health Literature (CINAHL) Complete (via EBSCO), Embase, MEDLINE (via EBSCO), Scopus, and Web of Science. In addition, Google Advanced Search and three grey literature databases were searched: OpenGrey, CareSearch grey literature, and ProQuest grey literature.

An initial search of CINAHL Complete (via EBSCO) was undertaken with a consulting librarian to identify key terms, keywords, and index terms. Keywords and terms about palliative care patients, PI risk assessment tools and acute hospitals were used to capture all relevant articles about the topic of interest. The terminology ‘acute palliative care patients’ is not yet prevalent in the existing literature and is not evidenced as a specific MeSH heading. To ensure the broadest sample of relevant data was captured for this scoping review broader terms such as ‘palliative care’, ‘palliative medicine’ alongside search terms such as ‘hospital’ and ‘acute care’ were used.

Variations of the exact words were used by combining descriptors, including subject headings and subject terms for each database, using free-text and truncated terms (“*”) to enhance the search. The search function Boolean operators (AND, OR) were used to combine terms.

In March 2023, the final search of the databases, grey literature and Google Advanced Search was undertaken. Table 2 presents the search strategy for CINAHL Complete via EBSCO*host*.

**Table 2:** Search strategy for CINAHL complete via EBSCO*host*

Search Query	
# 1	AB ( “pressure injur*” OR “pressure sore*” OR “pressure ulcer*” OR “pressure wound*” OR “bed sore*” OR bedsore* OR “bed ulcer*” OR “decubitus ulcer*” OR “decubitus sore*” OR “decubitus wound*” OR nurs*) OR TI ( “pressure injur*” OR “pressure sore*” OR “pressure ulcer*” OR “pressure wound*” OR “bed sore*” OR bedsore* OR “bed ulcer*” OR “decubitus ulcer*” OR “decubitus sore*” OR “decubitus wound*” OR nurs*)
# 2	(MH “Pressure Ulcer”)
# 3	# 1 OR # 2
# 4	AB (scor* OR scale* OR instrument* OR risk assess* OR assess* OR Braden OR “Hunters Hill” OR Norton OR Waterlow OR Walsall OR “clinical assessment*” OR “clinical judg*” OR “nurs* assess*”) OR TI (scor* OR scale* OR instrument* OR risk assess* OR assess* OR Braden OR “Hunters Hill” OR Norton OR Waterlow OR Walsall OR “clinical assessment*” OR “clinical judg*” OR “nurs* assess*”)
# 5	(MH “Clinical Assessment Tools”) OR (MH “Braden Scale for Predicting Pressure Sore Risk”) OR (MH “Gosnell Pressure Score Risk Assessment Instrument”) OR (MH “Pressure Ulcer Prevention (Iowa NIC)”) OR (MH “Risk Assessment”) OR (MH “Risk for Impaired Skin Integrity (NANDA) “) OR (MH “Nursing Assessment”)
# 6	# 4 OR # 5
# 7	AB (palliative OR hospital* OR patient*) OR TI (palliative OR hospital* OR patient*)
# 8	(MH “Palliative Care”) OR (MH “Palliative Medicine”) OR (MH “Adverse Health Care Event”) OR (MH “Health Facilities”) OR (MH “Hospital Units”) OR (MH “Hospitals”) OR (MH “Iatrogenic Disease”) OR (MH “Inpatients”) OR (MH “Hospitalization”) OR (MH “Acute Care”) OR (MH “Secondary Health Care”) OR (MH “Tertiary Health Care”)
# 9	# 7 OR # 8
# 10	# 3 AND # 6 AND # 9

Search conducted in March 2023

### Selection and eligibility of articles

The search results were imported into EndNote X9™ (Clarivate™). Citations were then transferred into Covidence™ (www.covidence.org), a web-based software designed to screen references and undertake data extraction for systematic and scoping reviews. The article selection phase in Covidence™ included title and abstract screening and full-text review. Two reviewers worked independently for each step, with a third reviewer acting as an arbitrator.

Title and abstract screening in Covidence™ involved two reviewers independently screening the article titles and abstracts against the predefined inclusion and exclusion criteria. Articles that potentially met the inclusion criteria based on title and abstract review were retrieved in full text. Where reviewers had any uncertainties about the relevance of a study or if the abstract was unclear, the full article was retrieved and assessed in detail against the inclusion criteria. Reference lists from the included articles were scrutinised to identify any potentially eligible citations not yet captured, their titles and abstracts were screened and, where relevant, included for full-text review [37]. Full-text studies that did not meet the inclusion criteria were excluded with reasons recorded.

Critical appraisal of included studies was not conducted because the aim of this scoping review was not to confirm or refute current practice based on current evidence or address uncertainty in practice [38].

### Data extraction and analysis

The initial data extraction template was guided by the review questions and PCC criteria [28]. An extraction table was created in Covidence™ and a pilot test of data extraction undertaken. Two reviewers performed data extraction independently. A third reviewer resolved disagreements that arose. Discussions between the reviewers occurred when further clarity was needed to achieve consensus. Data were exported from Covidence™ into Excel™ for narrative synthesis.

## Results

### Selection of articles

**Fig. 1:**
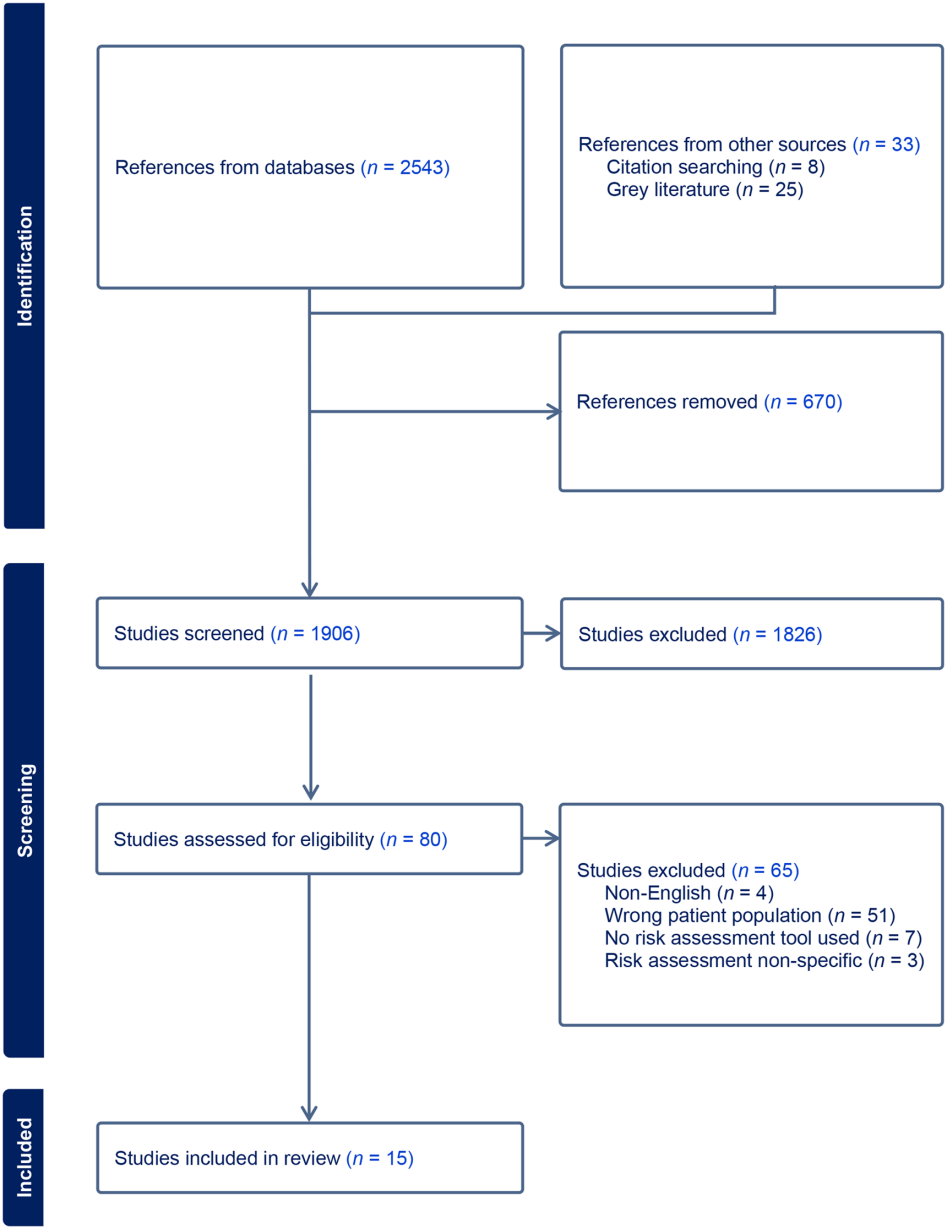
PRISMA-ScR

The initial search yielded 2,576 articles, from which 670 duplicates were removed. Of the 1,906 remaining articles screened by title and abstract, 80 advanced to full-text review. Full-text review resulted in the inclusion of 15 articles [15, 39–52]. See Fig. 1.

During the full-text screening process, it became evident that there was a lack of evidence using the language of ‘acute palliative care’ despite reporting on this cohort of patients. Consequently, studies reporting on palliative care with subset data applicable to the ‘acute palliative care’ cohort were included, with data related to EoL cohorts excluded during the data extraction phase.

### Article characteristics

The articles were published between 2002 and 2023. Over half (60%, *n* = 9) were from Europe, with a further third (33%, *n* = 5) from North America. Articles were set in a variety of clinical contexts, including specialist palliative care departments or units [42, 50, 51], hospice settings non-specific to terminal or EoL patients [40, 45, 46], inpatient hospitals [44, 49], cancer centres [41, 43], and non-specific palliative care settings [15, 39, 47, 48, 52].

Six primary studies were included [41, 45, 46, 48–50]. Of these, one study assessed the predictive validity and reliability of a new Curie scale [41], and another validity study described the correlation between the Braden Scale and the Palliative Performance Scale [48]. Jakobsen et al. [46] reported incidence and prevalence and evaluated risk factors associated with PI in a cohort study, and Sopata et al. [50] undertook an observational study to assess the influences of prophylaxis on PI development, comparing two risk assessment tools [Norton Scale and CBO scale (Dutch Consensus Prevention of Bedsores)]. A questionnaire survey was used by McGill and Chaplin [49] to describe prevention policies and identify risk assessment tools. A quality improvement study [45] compared risk factors of five risk assessments to construct a new scale and reported validity and reliability based on the scale’s predictions for patients with and without PI.

Nine articles were secondary studies using existing data sources [15, 39, 40, 42–44, 47, 51, 52]. Four audits were included [42–44, 51], of which two were retrospective audits of reported PI incidence and prevalence [42, 44]. Two articles described retrospective clinical chart reviews, with Sternal et al. [51] reporting risk factors as indicators of PI risk and Guo et al. [43] reporting the association between the Braden Scale and risk factors with overall-survival (OS) time for cancer patients.

Two articles were described as systematic reviews. Antony et al. [39] included 11 articles in their review of clinical practice guidelines for caregivers on the prevention of PI, and Ferris et al. [15] included 12 articles in their review of prevalence and incidence. The remaining three articles included a discussion on palliative wound care principles [40], guidelines and recommendations on the prevention and care of PI in palliative care patients [47], and a review of PI prevention strategy literature, which included 14 articles [52].

Seven articles reported the population as palliative care [15, 39, 40, 42, 47–49] with all authors used the broad term *palliative care* to describe all individuals, patients, or persons receiving palliative care. Six articles described an advanced cancer (oncology) population [41, 43–46, 50]. Various terms were used to describe the patient cohorts, including terminal illness (cancer), patients hospitalised for wound care in oncology, and cancer centre inpatients. Two articles used the term *advanced illness* to describe populations with non-specific diseases [51, 52]. Most articles described older adult samples aged 60 or more. Several articles describe an adult population but did not define specific characteristics [40, 47, 49, 52]. All study characteristics are detailed in Supplementary File 1.

### Pressure injury risk assessment tools

None of the included articles reported, discussed, or commented specifically on PI risk assessment tools for use with an acute palliative care patient cohort. However, all articles reported the use of at least one type of assessment tool to assess PI risk in palliative care patients. In total, 13 different PI risk assessment tools were identified. Four tools were reported in several articles, with the most common being the Braden Scale, which was reported in ten articles [39–41, 43, 45–49, 52]. The Waterlow Score was reported in five articles [15, 42, 45, 49, 51], the Norton Scale in four [41, 47, 49, 50], and the Hunters Hill risk assessment tool in two [47, 49]. Six articles reported the use of more than one of these tools [41, 45, 47, 49, 50, 52]. The various reported methods of assessment used to assess PI risk (directly and indirectly) are summarised in Table 3.

**Table 3:** Clinical assessment methods used to assess pressure injury risk

Assessment tools or methods	Description	Article sources
Braden Scale	The Braden Scale, also referred to as Braden Scale for Predicting Pressure Sore Risk, is a risk assessment tool that comprises six sub-scales (sensory perception, mobility, activity, moisture, nutrition, friction/shear). Developed in 1987 by Bergstrom et al. in the United States for older patients in a nursing home setting. The tool score is based on the sub-scale, with a low score representing a high-level PI risk.	Antony et al. [39]; Emmons et al. [40]; Fromantin et al. [41]; Guo et al. [43]; Henoch and Gustafsson [45]; Jakobsen et al. [46]; Langemo et al. [47]; Maida et al. [48]; McGill and Chaplin [49]; White-Chu and Reddy [52]
Waterlow Score	Developed in the United Kingdom in 1985 by Judy Waterlow for use in medical and surgical units. A scoring system calculates the score against several risk factors (appetite, sex/age, BMI, continence, skin type, mobility, tissue malnutrition, neurological deficit, major surgery/trauma, medication). A high score is associated with a high risk for PI development.	Ferris et al. [15]; Galvin [42]; Henoch and Gustafsson [45]; McGill and Chaplin [49]; Sternal et al. [51]
Norton Scale	Also referred to as the Norton scoring system, this tool was developed by Norton and colleagues in 1962. This tool is known to be one of the first tools to be developed to assess PI risk. A low score indicates a high risk for PI development. The scale comprises five assessment factors (general physical condition, mental state, activity, mobility, and continence.	Fromantin et al. [41]; Langemo et al. [47]; McGill and Chaplin [49]; Sopata et al. [50]
Karnofsky Performance Scale Index	Also referred to as the Karnofsky scale, this tool measures a patient’s performance status. The score is graded in percentages from 10-100%, where 10% is a comatose state or unconscious and 100% refers to a patient with full function. According to Hendrichova et al. [44], performance status lower than 50% suggests that the patient should be considered at high risk of developing a PI because for the Karnofsky Performance Scale a score of 40% indicates that the patient spends most of the time in bed, and a score of 30% indicates increasing debility and a requirement for total care.	Ferris et al. [15]; Hendrichova et al. [44]; Sopata et al. [50]
Hunters Hill Risk Assessment tool	Developed for inpatient palliative care patients at the Marie Curie Hospice in England in the year 2000, this tool is based on the Waterlow Score and Braden score risk assessment tools. The tool consists of seven factors believed to be specific for palliative care patients: sensation, mobility, moisture, activity in bed, nutrition/weight change, skin condition, friction/shear. A high score indicates a high risk for PI development.	Langemo et al. [47]; McGill and Chaplin [49]
Palliative Performance Scale	The Palliative Performance Scale is a common palliative care assessment and prognostication tool for survival for those patients with advanced illness and who are transiting to supportive and palliative care. The Palliative Performance Scale has been reported to have a positive correlation with the Braden Scale, thus, the authors suggested the Palliative Performance Scale may be used as a proxy for the Braden Scale in advanced illness patients.	Maida et al. [48]; White-Chu and Reddy [52]
Barthel Index	This functional assessment is used for palliative care patients with advanced chronic illness. The authors did not provide further details about the Barthel index assessment, only reporting that the Barthel index is used alongside the Waterlow Score at admission.	Sternal et al. [51]
Body mass index	BMI was reported to determine whether the variable influenced the onset of new PI.	Jakobsen et al. [46]
Clinical judgement	Clinical guideline recommendations for ‘clinical judgement’ to be used in conjunction with either a PI risk assessment tool specific for palliative care patients, a general screening tool, or other age-appropriate tools.	Langemo et al. [47]
CBO scale (Dutch Consensus Prevention of Bedsores)	Used to assess the degree of PI risk (low, moderate, high). Developed at the Academic Hospital in Utrecht, Netherlands, comprising 10 items (mental status, neurology, mobility, nutritional status, nutritional intake, incontinence, age, temperature, medication, diabetes mellitus). The CBO score ranges from 0 (low risk) to 2 points (high risk). The higher the sum, the higher the risk of developing a PI.	Sopata et al. [5]
Chaplin scale	A PI risk assessment scale for palliative care patients. The scale consists of eight risk factor items (Physical activity, Mobility, Food intake/nutrition, Incontinence/moisture, Friction/shearing, Sensory perception, Weight/body constitution, Type of skin). Each risk factor item is scored to determine the level of risk for PI development. Although, the risk factor item ‘Weight/body constitution’ is clustered with the ‘Food intake/nutrition’ item. The possible score for being at risk of PI is between 7–28.	Henoch and Gustafsson [45]
Curie scale	The scale consists of six items (mobility, incontinence, nutrition, patient participation, moisture/shearing, tissue damage). A score is used for each item; however, the score range differs for certain items. A score of 0 to 4 can be selected for three scale items: Mobility, Incontinence; Patient participation. For the item Nutrition, the score for selection can be either 0,2,3,4. For moisture/shearing, a score of 0,1,2, or 4 can be selected. For the remaining item, tissue damage, the possible score for selection is 0,2,4,6,12. No further details provided on how the score translates to risk level.	Fromantin et al. [41]
Douglas risk assessment tool	This tool was developed shortly after the Norton Score for a medical unit. The development was based on the Norton Scale and included additional factors thought to be important in PI development, nutritional status, pain, and skin condition. The tool associates a high score with a low level of risk.	McGill and Chaplin [49]
Hospice Pressure Ulcer Risk Assessment Scale	The scale was developed for palliative care patients in a Swedish hospice setting in 2003. The assessment items are physical activity and age. A score for ‘Physical activity’ and ‘Mobility’ is graded from 1 to 4, where 4 indicates full function and 1 indicates very deteriorated or no function. For ‘Age’, age below 75 years is graded as 2, while age ≥ 75 years is graded as 0. The possible score ranges from 2–10.	Henoch and Gustafsson [45]
Modified Norton Scale	The Norton Scale was revised by Ek et al. in 1989 in Sweden and consists of seven risk factors (mental state, physical activity, mobility, food intake/nutrition, fluid intake, incontinence/moisture, general physical condition). The score range for each risk factor is 1–4, for example, 1 indicates lack of function and 4 indicates normal function. A total score ≤ 20 indicates ‘at risk’ for PI development.	Henoch and Gustafsson [45]
Pressure Sore Risk Assessment Scale for Palliative Care	This scale was reported in review, however, details regarding the assessment or features of this scale was not provided by the authors.	White-Chu and Reddy [52]
Pressure Ulcer Scale in Oncology	The scale was developed for the Oncology population and validated by the developers in 2009. The creation of the Pressure Ulcer Scale Oncology was to simplify the Curie Scale. The Pressure Ulcer Scale Oncology consists of only three items: Bedridden/chair-ridden, incontinence, moisture/shearing. The authors reported these three items as key predictive factors for the development of PIs in cancer patients.	Fromantin et al. [41]
The Risk Assessment Pressure Sore scale	The RAPS scale was developed in Sweden by Ek and Lindgren in 1997. Based on the Braden Scale, the Norton Scale, the Modified Norton Scale, and the Waterlow Score. RAPS scale includes 13 risk factors: physical activity, mobility, food intake/nutrition, fluid intake, incontinence/moisture, general physical condition, fractioning/shearing, sensory perception, weight/body constitution, type of skin, body temperature, serum-albumin, blood pressure. A total sum score of ≥ 22 represents ‘extremely high risk’ for PI development.	Henoch and Gustafsson [45]
Pain assessment	Two pain assessment tools were reported as potential risk predictors for PI. (1) The Numerical Scale, and (2) Pain Assessment in Advanced Dementia Scale were assessments used as a predictor for potential PI risk. No further details were provided by the authors.	Jakobsen et al. [46]
Skin assessment	Reported that prevention of PI should include skin assessment. The authors included in the evidenced-based guidelines recommended that skin assessment be conducted as soon as possible (within a maximum of 4 h).	Antony et al. [39]

BMI: Body mass index, PI: Pressure injury, RAPS: Risk Assessment Pressure Sore

### Indirect assessment of pressure injury risk

Several assessment tools designed specifically for palliative care were reported as being used to assess PI risk. The use of the Karnofsky Performance Scale was reported in three articles [15, 44, 50], and the Palliative Performance Scale was reported as a proxy to assess PI risk by Maida et al. [48] and White-Chu and Reddy [52]. Several other methods were used in conjunction with PI risk assessment: the Barthel Index, a functional assessment to measure the patient’s ability to carry out activities of daily living [51]; body mass index to assess weight loss [46]; clinicians’ clinical judgement alongside a validated risk assessment tool to assess the risk for PI development [47]; skin assessment, part of a comprehensive assessment plan which included skin inspection and the Braden Scale [39]; and pain assessment in relation to the potential development of PI using tools such as the Numeric Rating Scale (NRS) and Pain Assessment In Advanced Dementia (PAINAD) scale [46].

### Patient characteristics and risk factors associated with pressure injury development

The characteristics of patients and risk factors associated with PI development are shown in Table 4. The majority of the articles reported patient age [15, 39, 41–46, 48, 50, 51], which ranged from 17 to 103 years, with one article including only adult (≥ 18 years) patients [39]. Of the 15 included articles, six articles reported the characteristics of patients who developed PIs while hospitalised [42–46, 51]. Of these, five articles reported the average age, which ranged from 62 to 79 years [42–45, 51]. Gender was reported in five articles [43–46, 51]. With the exception of one article [45] with a relatively small sample size (*n* = 20), gender distribution was similar.

**Table 4:** Summary of results for scoping review questions; (1) risk assessment to assess PI risk, (2) patient characteristics and risk factors associated with pressure injury development

Authors	Pressure injury risk assessment, Other clinical assessments	Characteristics of patients with PI	Risk factors
Antony et al. [39]	Pressure injury risk assessment tool: • Baden Scale Other clinical assessments for PI risk: • Conduct skin assessment as soon as possible (within 4 h of reaching home) • Comprehensive skin assessment twice daily	• Not reported	• Age • Increased body temperature • Anti-cancer drugs • General health status • Poor nutritional status • Oedema • Tissue consistency
Emmons et al. [40]	Pressure injury risk assessment tool: • Baden Scale for Predicting Sore Risk Other clinical assessments for PI risk: • None reported	• Not reported	• Not reported
Ferris et al. [15]	Pressure injury risk assessment tool: • Waterlow Score Other clinical assessments for PI risk: • None reported	• Not reported	• Age (*p* < 0.05) • Length of stay (*p* < 0.05) • Mobility (*p* < 0.05) • Waterlow score > 15 (*p* < 0.05)
Fromantin et al. [41]	Pressure injury risk assessment tool: • Braden Scale • Norton Scale • Curie scale • Pressure Ulcer Scale Oncology Other clinical assessments for PI risk: • None reported	• Not reported	• Age: mean 64.2 (SD 13.1) years, median63 (*p* = 0.02) • Increased body temperature/fever: >38 °C (*p =* 0.04) • Palliative/terminal admission: 24% (*p* = 0.003)
Galvin [42]	Pressure injury risk assessment tool: • Waterlow Score Other clinical assessments for PI risk: • None reported	*2000* • Average age: 65 years • Average length of stay: 24 days • Waterlow score 0–9 Not at risk: 6.4% (*n* = 2) 10–14 At risk: 6.4% (*n* = 2) 15–19 High risk: 32.2% (*n* = 10) 20 + Very high risk: 61.2% (*n* = 19) *2001* • Average age: 73 years • Average length of stay: 29 days • Waterlow score 0–9 Not at risk: 0 10–14 At risk: 2.9% (*n* = 1) 15–19 High risk: 29.4% (*n* = 10) 20 + Very high risk: 67.6% (*n* = 23)	• Age • Female gender • Length of stay
Guo et al. [43]	Pressure injury risk assessment tool: • Braden Scale Other clinical assessments for PI risk: • None reported	• Mean age 62.3 (SD 13.5) • Gender: female 42.9% (*n* = 191) • Ethnicity: % (*n*) Asian: 1.3 (6) Black: 10.3 (46) Hispanic 10.6 (47) Other 4.0 (18) Unknown 3.4 (15) White 70.3 (313) • Cancer diagnosis: % (*n*) Breast cancer: 3.6 (16) Brain and spinal: 2.7 (12) Gastrointestinal: 14.6 (65) Genitourinary: 10.1 (45) Gynaecological: 4.2 (19) Head and neck: 7.2 (32) Leukemia: 24.7 (110) Lung: 11.2 (50) Lymphoma: 6.5 (29) Melanoma: 2.7 (12) Multiple myeloma: 4.5 (20) No cancer: 0.4 (2) Sarcoma: 5.8 (26) Skin: 1.1 (5) Unknown primary: 0.4 (2) • Advanced stage: 77.9% (*n* = 345) • Mean body mass index: 28.5 (SD 8.3) • Pressure injury stage: % (*n*) 1: 18.4 (82) 2: 22.2 (99) 3: 6.1 (27) 4: 0.7 (3) Deep tissue injury: 37.3 (166) Unstageable: 9.0 (40) Unknown: 6.3 (28) • Pressure injury location % (*n*) Foot: 13.9 (62) Sacral/coccygeal area: 52.6 (234) Other: 29.7 (132) Unknown: 3.8 (17) • Braden score: % (*n*) ≤ 14: 53.0 (236) 15–18: 33.3 (148) 19–23: 13.7 (61)	• Age • Braden score • Mental alertness • Mobility • Neurological deficits in sensation • Poor nutritional status
Hendrichova et al. [44]	Pressure injury risk assessment tool: • Braden Scale Other clinical assessments for PI risk: • Karnofsky Performance Scale	• Mean age (years): 79 • Gender: male 48.1% (*n* = 13) • Karnofsky Performance Scale Index: % (*n*) 10: 0 20: 4 (1) 30: 67 (18) 40: 26 (7) 50: 4 (1) • Mean length of stay (days): 57 • Cancer diagnoses % (*n*) Head and neck cancer: 7 (2) Thorax cancer: 26 (7) Gastro-intestinal cancer: 41 (11) Breast cancer: 4 (1) Urogenital cancer: 15 (4) Haematological malignancies: 0 Other: 7 (2)	• Age: 79.9 (SD 6.8) (*p* < 0.0001) • Karnofsky Performance Scale Index (*p* < 0.001) • Length of stay: 57.2 days (*p =* 0.027)
Henoch and Gustafsson [45]	Pressure injury risk assessment tool: • Modified Norton Scale • Braden Scale • Waterlow Score • RAPS • Hospice Pressure Ulcer Risk Assessment Scale Other clinical assessments for PI risk: • None reported	• Mean age (years): 70.21 (SD 13.6) • Male 30% (*n* = 6) • Median length of hospice stay (days) 50.25 (range 7-263) • Paralysis or intradural anaesthesia *n* = 4 • Steroids *n* = 11 • Anti-inflammatory drugs *n* = 6 • Opioids *n* = 14	• Age (*p* < 0.023) • Mobility (*p* < 0.001) • Physical inactivity (*p* < 0.001)
Jakobsen et al. [46]	Pressure injury risk assessment tool: • Braden Scale Other clinical assessments for PI risk: • Body mass index	• Median Braden score 15 (IQR 15.5) • Pain valuated with numeric rating scale • Male 52% (*n* = 39) • Diagnosis primary tumour: % (*n*) Brain and nervous system 18.2 (2) Haematological: 20.0 (6) Hepatic-bile-pancreatic: 18.3 (15) Gastrointestinal: 18.3 (15) Genitourinary: 13.4 (11) Breast 10.5 (3) Skin (melanoma): 14.3 (1) Pulmonary: 18.5 (20) Head and neck: 10.0 (2) Other: 16.7 (1) • Sedation: 0 • Fractures: 11.1 (2) • Obligatory position: 15.0 (12) • State of consciousness • Cachexia: 17.9% (*n* = 45) Body mass index 18.5–24.9: 19.4% (*n* = 27) Dyspnoea: 19.9% (*n* = 46) • Lotion: 15.1% (*n =* 24) • Mattresses Polyurethane foam: 19.9% (*n* = 46) Dynamic air pressure Relieving: 15.1% (*n* = 24) Other types of mattress: 13.9% (*n* = 5)	• Age (mean): 76.4 years (PI) vs. 73.2 (non-PI) (*p =* 0.002) • Median days of recovery: 21.5 (PI) vs. 13 (non-PI) (*p <* 0.0001)
Langemo et al. [47]	Pressure injury risk assessment tool: • Braden Scale • Hunters Hill Marie Curie Centre Risk Assessment Tool • Norton Scale Other clinical assessments for PI risk: • Clinical judgement	• Not reported	• Age • Mobility • Friction/shearing • Moisture • Protein-calorie malnutrition
Maida et al. [48]	Pressure injury risk assessment tool: • Braden Scale Other clinical assessments for PI risk: • Palliative Performance Scale	• Not reported	• Age • Braden score: score ≤ 9 • Palliative Performance Scale: score 10-20%
McGill and Chaplin [49]	Pressure injury risk assessment tool: • Braden Scale • Douglas • Hunters Hill risk assessment • Norton Scale • Waterlow Score Other clinical assessments for PI risk: • None reported	• Not reported	• Not reported
Sopata et al. [50]	Pressure injury risk assessment tool: • Norton Scale • CBO scale Other clinical assessments for PI risk: • None reported	• Not reported	• Not reported
Sternal et al. [51]	Pressure injury risk assessment tool: • Waterlow Score Other clinical assessments for PI risk: • None reported	• Mean age (years): 71.5 (SD 11.4) • Female: 56.4% • Lung cancer patients: 15.4% • Colorectal cancer patients: 28.2% • Breast cancer patients 5.1% • Brain tumour patients: 0 • Stomach cancer patients: 10.3% • Disseminated cancer of unknown origin patients: 2.6% • Non-oncological patients with advanced heart failure: 0 • Patients with coexisting dementia: 17.9% • Patients with coexisting hypertension:46.2% • Patients with coexisting diabetes: 20.5% • Direct transfer from the hospital: 56.4% • Mean preadmission nursing home residency (days): 13.1 (SD 32.2) • Mean systolic blood pressure at admission (mmHg): 111.2 (SD 16.4) • Mean systolic blood pressure (mmHg): 106.1 (SD 13.1) • Mean diastolic blood pressure at admission (mmHg): 66.8 (SD 11.5) • Mean diastolic blood pressure (mmHg): 64.0 (SD 9.0) • Mean evening body temperature (°C): 36.9 (SD 0.5) • Mean haemoglobin level at admission (g/dL): 10.4 (SD 2.4) • Mean lowest recorded haemoglobin level (g/dL): 9.8 (SD 2.4) • Mean highest recorded haemoglobin level (g/dL): 10.6 (SD 2.4) • Mean lowest recorded erythrocyte count (g/L): 3.4 (SD 0.8) • Mean white blood cell count at admission (g/L): 12.9 (SD 6.2) • Mean highest recorded white blood cell count (g/L): 14.0 (SD 7.0) • Mean sodium serum concentration at admission (mmol/L): 135.4 (SD 7.4) • Mean lowest recorded sodium concentration (mmol/L): 131.3 (SD 5.6) • Mean breakthrough pain attacks per day: 0.8 (SD 0.8) • Mean body mass reduction during last 6 months (kg): 3.1 (SD 5.0) • Mean onset of physical deterioration (weeks): 27.4 (SD 6.0) • Mean Waterlow score at admission: 27.4 (SD 6.0) • Mean Waterlow score: 28.6 (SD 4.9) • Mean Barthel index at admission: 23.2 (SD 16.5)	• Mean evening body temperature: OR 3.830, 95% CI 1.729–8.486 (*p* = 0.001) • Mean Waterlow score: OR 1.194, 95% CI 1.092–1.306 (*p <* 0.001) • Lowest recorded sodium concentration: OR 0.880, 95% CI 0.814–0.951 (*p* = 0.001) • Mean systolic blood pressure: OR 0.956, 95% CI 0.929–0.984 (*p* >= 0.003) • Lowest recorded haemoglobin level: OR = 0.803, 95% CI = 0.672–0.960 (*p* = 0.016)
White-Chu and Reddy [52]	Pressure injury risk assessment tool: • Braden Scale • Pressure Sore Risk Assessment Scale for palliative care Other clinical assessments for PI risk: • Palliative Performance Scale	• Not reported	• Not reported

PI: Pressure injury, RAPS: Risk Assessment Pressure Sore

In total, 25 PI risk factors were identified across 11 articles [15, 39, 41–48, 51]. In 10 articles, age was proposed as a main factor that was significantly associated with PI development [15, 39, 41–48]. Notably, five risk factors which are familiar in palliative care, advanced illness, or cancer patients, were reported to be strongly associated with PI development: anti-cancer drugs [39], increased body temperature, low sodium concentration, low systolic blood pressure [51], and protein-calorie malnutrition [47]. Additionally, palliative care-specific clinical assessment scores including low (< 30) Karnofsky Performance Scale Index [44] and low Palliative Performance Scale scores [48] were identified as factors for PI development. Four articles reported length of stay as a factor linked to PI development [15, 42, 44, 46]. The various risk factors reported to be associated with PI development are shown in Table 5.

**Table 5:** Risk factors associated with the development of pressure injury

Risk factors	Article sources
Age	Antony et al. [39]; Ferris et al. [15]; Fromantin et al. [41]; Galvin [42]; Guo et al. [43]; Hendrichova et al. [44]; Henoch and Gustafsson [45]; Jakobsen et al. [46]; Langemo et al. [47]; Maida et al. [48]
Anti-cancer drugs	Antony et al. [39]
Braden score	Guo et al. [43]; Maida et al. [48]
Female	Galvin [42]
Friction/shearing	Langemo et al. [47]
General health status	Antony et al. [39]
Haemoglobin level	Sternal et al. [51]
Evening body temperature	Sternal et al. [51]
Fever/increased body temperature > 38^o^c	Antony et al. [39]; Fromantin et al. [41]
Karnofsky Performance Scale Index	Hendrichova et al. [44]
Length of stay	Ferris et al. [15]; Galvin [42]; Hendrichova et al. [44]; Jakobsen et al. [46]
Low sodium concentration	Sternal et al. [51]
Low systolic blood pressure	Sternal et al. [51]
Mental alertness	Guo et al. [43]
Mobility	Ferris et al. [15]; Guo et al. [43]; Henoch and Gustafsson [45]; Langemo et al. [47]
Moisture	Langemo et al. [47]
Neurological deficits in sensation	Guo et al. [43]
Oedema	Antony et al. [39]
Poor nutritional status	Antony et al. [39]; Guo et al. [43]
Palliative Performance Scale	Maida et al. [48]
Palliative/Terminal admission	Fromantin et al. [41]
Physical inactivity	Henoch and Gustafsson [45]
Protein-calorie malnutrition	Langemo et al. [47]
Tissue consistency	Antony et al. [39]
Waterlow score	Ferris et al. [15]; Sternal et al. [51]

## Discussion

To our knowledge, this scoping review is the first to map the available evidence on PI risk assessment for acute palliative care patients. The results reveal heterogenous research designs and populations. Although several formal PI risk assessment tools and several other assessment methods were described concerning PI risk assessment, the results show that there is currently no PI risk assessment tool available that has been designed specifically to assess *acute* palliative care patients.

The most commonly reported PI risk assessment tool was the Braden Scale, a clinically validated risk assessment tool for acute medical settings [47], yet this review yielded no evidence to suggest it has been validated in the acute palliative care setting. In a home palliative setting, the Braden score for those *at risk* (OR 1.92; 95% CI 1.17–3.14) was highly significant (*p* < 0.001) as a predictive factor for new PI development [24, 53]. Similarly, the Braden score (OR 0.83; 95% CI 0.82–0.84) was a variable with strong association to 180-day mortality in EoL palliative patients [54]. When comparing the Braden Scale with other assessments, researchers reported a significant positive correlation (*r* = 0.885, *p* < 0.001) between the Braden score and the Palliative Performance Scale in advanced illness patients [48]. Likewise, the Braden Scale was used to validate the use of the Pressure Ulcer Scale Oncology to assess PI risk for cancer patients in both curative and palliative phases [41]. Together these findings offer some insight into the use of the Braden Scale across different palliative settings. However, the results were limited to non-hospital settings.

The Waterlow Score was also referred to in several studies. Galvin [42] claimed that the Waterlow Score predicted 95.3% of those in the high and very high-risk groups subsequently developed pressure damage. However, a review by Walsh and Dempsey [55] found that the Waterlow Score had poor interrater reliability. In their United Kingdom (UK) survey of palliative care inpatient units, McGill and Chaplin [49] found that the Waterlow Score was used by 71% of respondents whereas the Braden Scale was used by only 3%. However, a significant proportion (41%) of survey respondents criticised the Waterlow Score for having various problems, including that it was non-specific to palliative care. Similarly, in a validity study examining risk scales for the oncology population [41], the authors argued that the Waterlow Score was poorly adapted to oncology because it contained irrelevant items (although these were not stated). In that study, compared to the Curie scale, the Norton Scale outperformed the Waterlow Score.

Two articles reported use of the Hunters Hill risk assessment tool in an acute setting [47, 49]. This tool was designed by the Marie Curie Centre specifically for palliative care patients [56], yet the survey by McGill and Chaplin [49] found that it was used in only a very small portion (2%) of palliative care units. In an earlier study pre-dating this review, a similarly named tool (Hunters Hill Marie Curie Centre pressure sore risk assessment tool) was reported [56]. However, based on the tool items (sensation, mobility, moisture, activity in bed, nutrition/weight change, skin condition, friction/shear), it is likely to be the same instrument. This scoping review identified two other very similar tools: the Chaplin scale [45] and the Curie scale [41], but the number of items in each differs from that of the Hunters Hill tool. The Chaplin scale comprised eight items, whereas the Curie scale comprised six items. Nonetheless, their similarities suggest that the Chaplin scale and Curie scale are variations or earlier or modified versions of the Hunters Hill tool, and both cite Chaplin [56] as a reference for the tool, along with Langemo et al. [47] and McGill and Chaplin [49]. These variations highlight the need for authors of future studies to identify clearly which tool they are reporting, to avoid potential confusion for clinicians.

Several studies supported a population-specific scale for vulnerable patients, such as palliative care cohorts [45, 56]. Henoch and Gustafsson [45] developed the Hospice Pressure Ulcer Risk Assessment Scale for patients with advanced cancer in a palliative care setting by excluding several risk factors from the Modified Norton Scale, leaving only three factors: physical activity, mobility, and age. Similarly, the Curie scale was used by Fromantin et al. [41] to develop the Pressure Ulcer Scale Oncology for cancer patients by using multivariate logistic regression analysis to identify three PI predictive factors in cancer patients: bedridden/chair-ridden, incontinence, and moisture/shearing. In palliative care, it is not uncommon for functional status assessment tools to be used instead of traditional PI risk assessment tools. Hendrichova et al. [44] reported that the Palliative Performance Scale had a significant relationship with the Braden score. Similarly, Maida et al. [48] suggested that the Palliative Performance Scale could be used as a proxy measure for the Braden Scale for patients with advanced illness. Their study reported a significant correlation (*r* = 0.885, *p* < 0.001) between the Palliative Performance Scale and the Braden Scale after controlling factors such as age, gender, consult site, and diagnosis (cancer versus non-cancer).

Palliative care is a diverse and unique population [14, 22]. The pathological process and irreversibility of some symptoms increase the risk for PI development [14]. The lack of similar studies on PI assessment and prevention for palliative care makes comparing patient characteristics challenging [14]. Only six of the 15 articles reported characteristics of patients who developed PIs while hospitalised [42–46, 51]. Similar to other studies, characteristics such as age and gender were reported mostly for patients with PIs during hospitalisation [15, 42–46, 51]. Not surprisingly, the majority of palliative care patients (including cancer patients, and patients with advanced or terminal illnesses) who developed PIs were assessed as high risk or higher when using either the Braden Scale or the Waterlow Score [42, 43, 46, 51]. However, in this review, descriptions of the palliative population were unclear and no distinction was made between ‘acute’ and ‘EoL’ palliative care patients.

Individuals receiving palliative care are at increased risk for PI development [11] due to altered skin integrity, mobility issues, and chronic diseases [15]. In terms of risk factors for PI development, this review revealed that age was the main risk factor reported [15, 39, 41–48]. Most risk factors reported in articles in this review are usually included in PI risk assessments of general medical acute patients (e.g., age, nutrition, incontinence, mobility) [45, 49]. However, several risk factors specific to palliative patients were identified, such as anti-cancer drug therapy [39], higher body temperature [51], low haemoglobin level [51], low sodium concentration [51], protein-calorie malnutrition [47], and low systolic blood pressure [51]. As well, changes to the lymphatic system with consequent lymphoedema associated with cancer treatment, as well as other factors such as progressive immobility, may increase the risk of PI development [57].

Whilst there is a substantial body of literature around PI in palliative care, use of the term palliative care is often ambiguous. An important finding in this review was that none of the articles differentiated between acute palliative care and EoL patients, using terms such as *palliative care* or *palliative phase*, making it difficult to exclude references to PI management in the terminal phase. For example, Fromantin et al. [41] stated that patients were admitted for palliative or terminal phase care but did not differentiate between the two cohorts, with their discussion relating to EoL care rather than acute phase palliative care. In the review by White-Chu and Reddy [52], the authors described patients with advanced illness as patients transitioning from curative to supportive and palliative care in various settings (such as acute care, intensive care, long-term care, palliative care or hospices). And, in a systematic review of PI studies in palliative care, it was found that palliative care patients were not well-defined [15].

In the current literature around PI risk assessment, there is a lack of clearly defined terminology regarding palliative patient cohorts with respect to their health trajectory. Thus, in this review, in the absence of clear definitions, we were unable to determine an appropriate PI risk assessment tool for use with acute palliative care patients. This illustrates a gap in knowledge about PI risk assessment of these patients. As pointed out by Hampton [58], this could lead to conflicting aims in clinical care. Given that several specific risk factors have been identified in this review, further research is needed in this area.

Despite an emerging body of literature on PI and palliative care, this review demonstrated a dearth of evidence available to assess PI risk specifically for acute palliative care patients. Whilst there is some research around PI prevention for terminal palliative care patients, PI at EoL is associated with the inevitable physiologic changes of dying (i.e., reduced skin and soft tissue blood perfusion) [23] and in this context, is primarily unavoidable. In this context, nursing care is focused on comfort. This differs from palliative care in the acute phase, which focuses on improving QoL and preventing unnecessary harm to the patient, including avoidable PI. In summary, the currently available literature contributes little to the prevention and management of PI in acute palliative care patients.

### Limitations and strengths

This scoping review was limited to studies published in English; consequently, any relevant articles in other languages were not captured. Strengths of the review include using the PCC framework to rigorously select relevant articles. A transparent and rigorous approach was taken to conduct and report the review in accordance with the PRISMA-ScR guidelines [34]. Strengths also lie in including literature over the past 20 years and the multiple perspectives, expertise and experience researchers contributed to a detailed overview. Although this review adhered to an a priori protocol, a minor modification was the inclusion of palliative care-specific clinical assessments, included during the data extraction phase when it was found that instruments such as the Karnofsky Performance Scale and Palliative Performance Scale were reported as being used to assess the risk of PI.

## Conclusion

Palliative care patients are vulnerable to PI [39], and risk assessment is recommended to help prevent PI development [22]. The included articles reported 13 different risk assessment tools and seven palliative care clinical assessment methods used to assess PI development. However, this review found no articles that reported or discussed a PI risk assessment tool or method specifically for the acute palliative care patient cohort. Similarly, whilst most articles reported multiple patient characteristics and a variety of risk factors associated with PI development, none of the articles defined or described the patient population as acute palliative care. Nonetheless, these findings revealed a research gap and a need to contextualise acute palliative care in PI prevention research.

Furthermore, the reviewed articles lacked a clear definition of the palliative care cohort included in each article. The complexity of prevention and management of PI in acute palliative patients is primarily due to the lack of clear definitions of palliative care patients and the complete absence of a suitable risk assessment tool. Therefore, research is needed to develop and test a risk assessment tool specific to this cohort to ensure accurate assessment of risk factors and appropriate evaluation of PI risk. In turn, this should lead to the implementation of appropriate preventative interventions to mitigate risk.

## Electronic supplementary material

Below is the link to the electronic supplementary material.

Supplementary Material 1

## Data Availability

For copyright reasons, data used in this manuscript consist of published articles which cannot be shared by the authors but are available through subscription to the relevant journals or databases.

## Abbreviations

CINAHL: Cumulative Index to Nursing and Allied Health Literature
EoL: End-of-life
JBI: Joanna Briggs Institute
KTU: Kennedy Terminal Ulcer
NRS: Numeric Rating Scale
OS: Overall-survival
OSF: Open Science Framework
PAINAD: Pain Assessment In Advanced Dementia
PCC: Population, Concept, Context
PI: Pressure injury
PRISMA-ScR: Preferred Reporting Items for Systematic Reviews and Meta-Analyses for Scoping Reviews
QoL: Quality of life
SCALE: Skin Changes At Life’s End
UK: United Kingdom
