# Supplementary material for: Pressure injury risk assessment for palliative care patients in the acute hospital setting: a scoping review
Source: BMC Palliat Care. 2025 Jul 25;24:212. doi: 10.1186/s12904-025-01842-y (PMC12297692; doi:10.1186/s12904-025-01842-y)
Supplement: Supplementary file 1 — Supplementary Material 1 [file 12904_2025_1842_MOESM1_ESM.docx]

Summary of the included articles

| Author(s), year, country | Aims, methods | Population, sample size, age, gender, setting | Key findings |
| --- | --- | --- | --- |
| Antony et al., 2023, India [39] | *Aims*: primary: to implement evidence-based guidelines for caregivers of palliative care patients on PI prevention; secondary: to improve the knowledge and practice of caregivers and enable them to take measures to prevent PI development among palliative care patients.  *Methods:* systematic review of 11 articles for qualitative synthesis to prepare clinical practice guidelines for PI prevention. | *Population*: palliative care patients.  *Sample*: clinical practice guidelines *n =* 2; systematic reviews *n =* 4; randomised controlled trials (RCT) *n =* 5  *Age*: ≥ 18 years (inclusion criterion for studies)  *Gender*: not applicable  *Setting*: all settings with palliative care patients | - Following systematic review of the evidence, multiple clinical practice guidelines were recommended. Within the skin assessment and skincare guidelines, use of the Braden Scale was recommended. |
| Emmons et al., 2014, United States [40] | *Aim:* to discuss palliative wound care principles by common types of wounds and then practical management of specific symptoms regardless of wound aetiology.  *Methods*: discussion paper (Part 2 of 2-part series) on principles of palliative wound care. | *Population*: palliative care patients.  *Sample size*: not applicable  *Age*: not applicable  *Gender*: not applicable  *Setting*: hospice, home care setting | - The Braden Scale was the only tool that was proposed to assess PI risk in palliative care patients. - A prevention program based on the Braden Scale might consist of interventions focusing on each of the sub-scores but modified for palliative care patients. |
| Ferris et al., 2019, United Kingdom (Wales) [15] | *Aims*: to quantify the prevalence and incidence of PI in patients receiving palliative care and identify the risk factors for PI development in these patients as well as the temporal relationship between PI development and death.  *Methods*: systematic review of 12 articles looking at palliative care patients identified as having PIs. | *Population*: palliative care patients.  *Sample size: r*etrospective cohort *n =* 5; prospective cohort *n =* 4; retrospective case control *n =* 2; audit *n =* 1  *Age*: not applicable  *Gender*: not applicable  *Setting*: community, inpatient | - A high Waterlow Score was associated with PI development (score > 15 in 93% of patients with PI development). - Patients with a PI had an average Waterlow Score of 27.4. |
| Fromantin et al., 2011, France [41] | *Aim*: To assess the predictive validity and reliability of the new Curie scale for patients in oncology, with a view to how it could be improved or optimised.  *Methods*: Validity study – convergent *validity* to determine correlation. | *Population*: advanced cancer patients.  *Sample size:* 933 patients surveyed (2002 *n* = 351; 2009 *n* = 582)  *Median age*: 2002: 57 years (range 17-91); 2009: 60 years (range 16-89)  *Gender*: 2002: male 29% (*n* = 100); 2009:  male 25% (*n* = 148)  *Pressure Injury on admission:* 2002: 5% (*n* = 18); 2009: 5% (*n* = 31)  *Transfer from another hospital:* 2002: 7% (*n* = 25); 2009: 13% (*n* = 74)  *Reason for admission:* 2002: chemotherapy 19% (*n* = 67); surgery 47% (*n* = 163); radiotherapy 6% (*n* = 20); palliative 6% (*n* = 21); other 22% (*n* =  77); 2009: chemotherapy 14% (*n* = 83); surgery 50% (*n* = 288); radiotherapy 5% (*n* = 30); palliative 5% (*n* = 29); other 26% (*n* = 152)  *Aplasia*: 2002: 3% (*n* = 11); 2009: 3% (*n* = 19)  *Fever (> 38^o^C):* 2002: 8% (*n* = 27); 2009:  5% (*n* = 28)  *Corticosteroid therapy:* 2002: 32% (*n* = 113); 2009: 26% (*n* = 153)  *Setting:* wound care unit | - Curie scale: cut-off values of > 14 and 14 were identified for the Norton Scale, using Youden index, demonstrating a strong correlation with PI prevalence. - Only three individuals (1.3%) with a score > 14 developed a PI, while 15 (12.4%) with a score ≤ 14 developed a PI (*p* < 0.0001). Cut-offs for the Curie scale - values determined were 3 and > 3, with a PI prevalence rate of 0.4% for a Curie scale score 3 and 16.5% for a score > 3 (*p* < 0.0001). - A high score on the Curie scale (indicating PI risk) was correlated with a lower score on the Norton Scale (also indicating PI risk). The Curie scale, expressed according to the cutoffs 3 and > 3, gave a relatively high Kappa value of 0.69, which was highly significant (*p* < 0.0001). The Waterlow Score was found to be poorly adapted to oncology, containing many irrelevant items. - The Pressure Ulcer Scale Oncology: The Curie scale presented good agreement with the Braden Scale (Spearman rho 0.83, *p* < 0.001) for quantitative scores. The Curie scale, with cut-offs 3 and > 3 as defined earlier, again gave a Kappa value of 0.60, which was highly significant (*p* < 0.0001). There was a strong correlation between the Pressure Ulcer Scale Oncology score and the PI prevalence rate (*p* < 0.0001) |
| Galvin, 2002, United Kingdom [42] | *Aim*: To describe the implementation and findings of a PI audit monitoring the incidence of PI in a 16-bedded specialist palliative care inpatient unit over a 2-year period.  *Methods*: Retrospective audit of PI incidence within a specialist unit over 2 years. | *Population*: palliative care patients.  *Sample size: n* = 543  *Age*: average 68 (range 35-90)  *Gender*: mixed  *Setting*: specialist palliative care unit | - Over the 2-year period the Waterlow Score accurately predicted 95.3% of those who developed pressure damage as being in the high and very high-risk groups. |
| Guo et al., 2022, United States [43] | *Aim*: To assess risk factors associated with overall survival (OS) of hospitalised cancer patients with PI.  *Methods*: Retrospective review of patient chart. | *Population*: inpatients NCI-Designated Cancer Centre, referred to Wound Ostomy Continence Care nurse for PI.  *Sample size:* 445  *Mean age*: 62.3 (SD 13.5)  *Gender*: female 42.9% (*n* = 191)  *Setting*: cancer centres | - A lower Braden score was significantly associated with shorter OS of cancer patients with PI (*p* < 0.0001). Median survival times for patients with Braden score ≤ 14, 15-18, and ≥ 19 were 0.4 months (95% CI 0.4-0.6), 5.2 months (95% CI 3.0-9.2), and 8.4 months (95% CI 4.9-NA), respectively. - In a multivariate Cox model, adjusted for advanced disease, Braden score of 15-18 and ≥ 19 is shown to be significantly associated with longer OS time when compared with patients with Braden score ≤ 14 (HR 0.44, *p* < 0.0001; HR 0.47, *p* < 0.0001, respectively). |
| Hendrichova et al.,  2010, Italy [44] | *Aim*: To contribute to the knowledge base about pressure ulcers in palliative care through the description of prevalence and incidence of pressure ulcers in terminally ill cancer patients in an Italian palliative care service.  *Methods*: Descriptive study conducted using a retrospective analysis of clinical records. | *Population*: terminally ill cancer patients  *Sample size:* 414  *Mean age*: 74  *Gender*: male 49.7% (*n* = 206)  *Setting*: inpatient hospital | - In palliative care, performance status was measured with the Karnofsky Performance Scale Index or with the Palliative Performance Scale which were used interchangeably. Within palliative care, these scales were also commonly used instead of the Braden Scale, a measure specifically aimed at evaluating the risk of developing PIs, as the Palliative Performance Scale shows a significant relationship with the Braden score. Scores of the Karnofsky Performance Scale index were distributed with significant difference (*p* < 0.001) between patients who had PI when admitted and those without PIs at admission. The Karnofsky Performance Scale indicates 20% and 30% were frequent inpatients with PIs at admission. |
| Henoch and Gustafsson, 2003, Sweden [45] | *Aims*: To compare the occurrence of risk factors from the modified Norton, the Braden, the Waterlow and the RAPS scales with the occurrence of pressure ulcers in palliative care. To construct scales for the assessment of pressure ulcer risk appropriate for use in palliative care using the results from the above comparisons. To determine the most appropriate scale for this group of patients by calculations of validity and significant differences in prediction scores between the patients with and without pressure ulcers when using the constructed scales.  *Methods*: quality improvement study to develop a hospice PU risk assessment scale. | *Population*: advanced cancer patients  *Sample size:* 98  *Age*: 26-95 years old  *Gender*: male 35.7% (*n* = 35)  *Setting*: hospice | - After the modified Norton Scale and the nine constructed scales were tested, only scale X showed both statistically significant differences between the groups of patients with and without PIs and had high validity both on admission and at time of development of PIs. Following these results scale X was named the Hospice Pressure Ulcer Risk Assessment Scale. This includes the following assessment items: physical activity, mobility, age. |
| Jakobsen et al., 2020, Italy [46] | *Aims*: to measure the incidence and prevalence of pressure ulcers in a Hospice environment; evaluate the risk factors associated with pressure ulcers; and calculate the incidence of Kennedy Terminal Pressure Ulcers.  *Methods*: Observational prospective cohort study. | *Population*: Cancer patients in advanced phase  *Sample size:* 440  *Age*: 73.7 years (range 37-98)  *Gender*: Male 51.4% (*n* = 226)  Female 48.6% (*n* = 214)  *Setting*: hospice | - The Braden score in patients who developed a new PI was not different to scores in the general population (*p* = 0.800). |
| Langemo et al., 2010, United States [47] | *Aim*: To review and summarise the current scientific evidence for prevention and care of a PrU in a palliative care patient.  *Methods*: Review article summarising evidence for prevention and care of pressure ulcer. | *Population*: individuals receiving palliative care.  *Sample size:* not reported  *Age*: not reported  *Gender*: not reported  *Setting*: non-specific | - Recommendation: to assess the risk for new PI development at the time of admission and on a regular basis in the patient receiving palliative care by using a validated risk assessment tool. - Recommendation: to use the Hunters Hill Marie Curie Centre Risk Assessment Tool, specific to the patient receiving palliative care, or a general screening tool, such as the Braden Scale, Norton Scale, or other age-appropriate tool, in conjunction with clinical judgment for the adult individual. |
| Maida et al., 2008, Canada [48] | *Aim*: not stated.  *Methods*: validity study describing the correlation between the Braden Scale and the Palliative Performance Scale. | *Population*: patients referred for supportive and palliative care in a combined community and hospital-based palliative medicine programme.  *Sample size:* 664  *Age*: mean 74.9 (range 19-103)  *Gender*: male 49.7% (*n* = 330)  *Setting*: community and hospital-based palliative medicine programme | - There was a strong linear correlation between Braden Scale and Palliative Performance Scale in the setting of patients with advanced illness referred for supportive and palliative care. A strong linear trend was shown between the initial Braden Scale and the initial Palliative Performance Scale scores. The significant correlation between Palliative Performance Scale and Braden Scale persisted whether the effect of other factors were removed or not (*r* = 0.936 before and *r* = 0.885 after; *p* < 0.001). |
| McGill and Chaplin, 2002, United Kingdom [49] | *Aims*: To determine current pressure ulcer prevention policy in palliative care inpatient units in the UK and to determine the elements included in pressure ulcer prevention policies and to identify which pressure ulcer risk assessment tools are used in palliative care inpatient units.  *Methods*: article reports part 1 of the results of a postal questionnaire survey. | *Population*: UK specialist palliative care inpatient units.  *Sample size*: 102/206 PCUs (completed survey). Response rate 49.5%  *Age*: not reported  *Gender*: not reported  *Setting*: inpatient hospital | - Waterlow was the most commonly reported PI risk assessment tool in use in palliative care inpatient units (71%). Other tools in use include Norton (5%), Braden (3%) and Douglas (3%); 4% of respondents indicated that they used no risk assessment tool, 2% used the Hunters Hill risk assessment tool. - The most commonly cited reasons for using the chosen tool were because it was: the most appropriate, familiar, or that it was the policy of the hospital/trust. 34% of all respondents identified problems in using their selected tool. A range of problems was identified with the Waterlow Score. The two most commonly cited were that it was non-specific to palliative care patients and that it tended to score most patients as high risk. |
| Sopata et al., 2014, Poland [50] | *Aims*: to assess the influence of prophylaxis on PI development in patients with advanced cancer. A comparative analysis of the risk assessment scales: the Norton Scale and the CBO scale (Dutch Consensus Prevention of Bedsores) was also conducted.  *Methods*: observational study. | *Population*: palliative care patients with advanced cancer  *Sample size:* 319  *Age*: mean 61 (SD 11.4, range 18-94)  *Gender*: male 60.5% (147/243)  *Mean hospitalisation:* 14 days  *ECOG on admission:* 3.5  *Karnosfsky scale on admission:* 40  *Main diagnosis: b*reast cancer: 22.6% (*n* = 88); genitourinary tract cancer 22.1% (*n* = 86); lung cancer 20.1% (*n* = 80); digestive tract cancer (19.5% (*n* = 76); other 15.4% (*n* = 60).  *Setting*: inpatient hospital, palliative care unit | - Pressure injury developed in 44 cases (13.2%) during admission. This means that the general effectiveness of the prophylactic interventions was 86.8%. Most pressure ulcers developed after the 15th day of hospitalization, as they appeared in 18 patients (40.9%). Then, they appeared in 16 patients (36.4%) between the 7th and 15th day, and in 10 patients (22.7%) before the 7th day of hospitalisation. - Two risk assessment scales were used in the group of 10 patients in whom PIs developed before the 7th day of hospitalisation, and their outputs were subsequently compared. The study showed no statistically significant difference between the scales in the observation period mentioned (*p* = 0.6250). The measurements of risk degree (high, moderate, low, none) show that the two scales are equivalent. - Two risk assessment scales were used in the group of 16 patients in whom PIs developed between the 7^th^ and 15^th^ day of hospitalisation, and their outputs were subsequently compared. No statistically significant difference was demonstrated (*p* = 0.0513). - High risk was shown by both scales in 18 patients in whom PI developed after the 15^th^ day of observation. This observation period also proved the scales to have equal sensitivity (NS; *p* = 0.5720). The comparative assessment of the degree of PI risk carried out by means of two scales Norton and CBO, demonstrated that, in all observation periods, both tools showed the same sensitivity. |
| Sternal et al., 2017, Poland [51] | *Aim*: to analyse factors associated with the development of PI in hospitalised patients with advanced illness.  *Methods*: retrospective analysis | *Population*: palliative care patients.  *Sample size:* 329  *Mean age*: age 70.4 (SD 11.8)  *Gender*: male 44.7% (*n* = 147)  *Setting*: inpatient hospital, palliative care department | - When assessed at admission, the Waterlow Score, was predictive of PI development (OR 1.140, 95% CI 1.057-1.229, *p* = 0.001). - During hospitalisation, the mean Waterlow Score was associated with PI development (OR 1.194, 95% CI 1.092-1.306, *p* < 0.001). |
| White-Chu and Reddy, 2013, United States [52] | *Aim*: to summarise the relevant recent literature addressing the prevention of PI in persons with advanced illness.  *Methods*: review summarising the literature from 1^st^ January 2011 to 1^st^ June 2012. | *Population*: persons with advanced illness  *Sample size:* 14 articles  *Age*: not reported  *Gender*: not reported  *Setting*: acute care, intensive care, long-term care, palliative care, hospice care. | - Of the 14 articles, only 4 articles included a risk assessment as a PI preventive strategy. One review supported the use of the Braden Scale or Pressure Sore Risk Assessment Scale for palliative care as quality predictors for PI development in the advanced illness population. One study reported a positive correlation between the Palliative Performance Scale and the Braden Scale. - The Palliative Performance Scale may be used as a proxy for the Braden Scale in patients in advanced illness. |

BMI: Body mass index, CI: confidence interval, OR: odds ratio, OS: Overall survival, PCU: Palliative care unit, PI: Pressure injury, PrU: Pressure ulcer, PU: Pressure ulcer, RAPS: Risk Assessment Pressure Sore, UK: United Kingdom
